# A Rapid Method for Detecting Normal or Modified Plant and Algal Carbonic Anhydrase Activity Using *Saccharomyces cerevisiae*

**DOI:** 10.3390/plants11141882

**Published:** 2022-07-20

**Authors:** Ashwani K. Rai, Robert J. DiMario, Remmy W. Kasili, Michael Groszmann, Asaph B. Cousins, David Donze, James V. Moroney

**Affiliations:** 1 Department of Biological Sciences, Louisiana State University, Baton Rouge, LA 70803, USA; arai5@lsu.edu (A.K.R.); rkasili@lsu.edu (R.W.K.); ddonze@lsu.edu (D.D.); 2 School of Biological Sciences, Washington State University, Pullman, WA 99164, USA; robert.dimario@wsu.edu (R.J.D.); acousins@wsu.edu (A.B.C.); 3 ARC Centre of Excellence in Translational Photosynthesis, Research School of Biology, Australian National University, Linnaeus Building, 134 Linnaeus Way, Canberra, ACT 2601, Australia; michael.groszmann@anu.edu.au

**Keywords:** carbonic anhydrase, CA activity, C_3_ plants, *S. cerevisiae*, Arabidopsis, protein expression, photosynthesis, MIMS

## Abstract

In recent years, researchers have attempted to improve photosynthesis by introducing components from cyanobacterial and algal CO_2_-concentrating mechanisms (CCMs) into terrestrial C_3_ plants. For these attempts to succeed, we need to understand the CCM components in more detail, especially carbonic anhydrase (CA) and bicarbonate (HCO_3_^−^) transporters. Heterologous complementation systems capable of detecting carbonic anhydrase activity (i.e., catalysis of the pH-dependent interconversion between CO_2_ and HCO_3_^−^) or active HCO_3_^−^ transport can be of great value in the process of introducing CCM components into terrestrial C_3_ plants. In this study, we generated a *Saccharomyces cerevisiae* CA knock-out (*ΔNCE103* or *ΔCA)* that has a high-CO_2_-dependent phenotype (5% (*v*/*v*) CO_2_ in air). CAs produce HCO_3_^−^ for anaplerotic pathways in *S. cerevisiae*; therefore, the unavailability of HCO_3_^−^ for neutral lipid biosynthesis is a limitation for the growth of *ΔCA* in ambient levels of CO_2_ (0.04% (*v*/*v*) CO_2_ in air).  *ΔCA* can be complemented for growth at ambient levels of CO_2_ by expressing a CA from human red blood cells. *ΔCA* was also successfully complemented for growth at ambient levels of CO_2_ through the expression of CAs from *Chlamydomonas reinhardtii* and *Arabidopsis thaliana*. The *ΔCA* strain is also useful for investigating the activity of modified CAs, allowing for quick screening of modified CAs before putting them into the plants. CA activity in the complemented *ΔCA* strains can be probed using the Wilbur–Anderson assay and by isotope exchange membrane-inlet mass spectrometry (MIMS). Other potential uses for this new *ΔCA-*based screening system are also discussed.

## 1. Introduction

Carbonic anhydrases (CAs) catalyze the interconversion between CO_2_ and bicarbonate (HCO_3_^−^) in solutions [1]. Although the interconversion of CO_2_ and HCO_3_^−^ happens without a CA, it occurs at a very slow rate. CAs are essential for organisms to ensure they have a quick supply of CO_2_ and HCO_3_^−^ for various metabolic pathways. CAs also play a crucial role in photosynthesis. For example, the CO_2_-concentrating mechanisms (CCMs) of *Chlamydomonas reinhardtii* and cyanobacteria are powered by CAs [2,3,4,5]. In the biophysical CCMs of cyanobacteria and green algae, ribulose 1,5-bisphosphate carboxylase/oxygenase (Rubisco) is packaged in very specific compartments—carboxysomes for cyanobacteria and pyrenoids for green algae. The CCMs work to accumulate HCO_3_^−^ to high levels in the cytosol (cyanobacteria) or chloroplast stroma (eukaryotic algae); then a specific CA is needed to convert the HCO_3_^−^ to CO_2_ for photosynthesis. This creates a local environment around Rubisco that has an elevated CO_2_ concentration. In *C. reinhardtii*, the conversion of HCO_3_^−^ to CO_2_ is catalyzed by CAH3 in the thylakoid lumen inside the pyrenoid. Loss of this thylakoid CAH3 in *C. reinhardtii* results in very slow growth rates at ambient levels of CO_2_ (~0.04% (*v*/*v*) CO_2_ in air) [6]. Similarly, carboxysomal CAs in cyanobacteria are required for the conversion of accumulated HCO_3_^−^ to CO_2_ for fixation by Rubisco [7]. For photosynthetic organisms to function efficiently, the CAs must be in the correct inter- and intracellular locations. For example, cyanobacterial CAs inside the carboxysomes are critical for maintaining the CCM, but CA activity in the cytoplasm disrupts the CCM. Price et al. [8] showed that CA expression in the cytoplasm of *Synechocystis* cells caused the CCM to short-circuit.

In C_4_ plants, the CCM is maintained by CA activity in mesophyll cells [9]. For C_4_ plants, the first step of photosynthesis is the conversion of CO_2_ that diffuses into the leaf mesophyll cells to HCO_3_^−^, which is catalyzed by a cytosolic CA [9]. DiMario et al. [10] demonstrated that the elimination of mesophyll cytoplasmic CA activity causes a reduction in photosynthesis for C_4_ plants grown in ambient levels of CO_2_. C_3_ plants, in contrast, do not have a CCM. However, C_3_ plants still have a large number of genes encoding CA. In Arabidopsis, the α, β, γ, and γ-like isoforms of CA are encoded by 17 distinct genes [2]. The role of CAs in terrestrial C_3_ plants is not well understood due to the compensatory effect of multiple isoforms. For example, DiMario, et al. [11] investigated the effects of knocking out the Arabidopsis CAs βCA2 and βCA4, which are present in the cytosol of leaf mesophyll cells, and observed that eliminating only one of the CAs resulted in no observable phenotype. When both βCA2 and βCA4 were knocked out, plants were unable to grow normally in low-CO_2_ conditions. In addition, Medina-Puche et al. [12] and Hines et al. [13] observed that single knock-out lines for most βCAs in Arabidopsis had normal growth on air.

There have been attempts in recent years to improve photosynthesis by introducing CCM components from cyanobacteria, algae, or C_4_ plants into terrestrial C_3_ plants [14,15,16]. For these approaches to work, CAs must be modified and retargeted to specific locations in C_3_ plants. This requires targeting CAs to the chloroplast thylakoid lumen or the cell wall. The CAs need to be modified and tagged to determine whether they are being targeted to the correct intracellular location. Thus, a rapid screen is needed to determine whether a protein modification inhibits CA activity. Transforming prospective CAs into plants is possible but requires significant time and resources. Heterologous complementation systems capable of detecting CA activity or active bicarbonate transport are valuable when studying CCM components in order to transform them into plants.

Here, we determined whether the high-CO_2_-dependent *Saccharomyces cerevisiae* CA knock-out line, *ΔNCE103* (referred to here as *ΔCA*), is suitable as a heterologous complementation system for the detection of active CAs from plants and algae. The *ΔCA* strain cannot grow on ambient levels of CO_2_ but can grow on high levels of CO_2_ (5% CO_2_ (*v*/*v*) in the air). The *ΔCA* strain lacks the gene *NCE103*, which encodes a single native *S. cerevisiae* CA [17]. It has been proposed that the loss of this native CA results in *S. cerevisiae* cells that do not have enough HCO_3_^−^ for important metabolic processes such as fatty acid and nucleotide synthesis [18]. It has been speculated that *S. cerevisiae* requires some CA activity for survival at ambient levels of CO_2_ because the uncatalyzed rate of CO_2_ hydration to HCO_3_^−^ produces insufficient HCO_3_^−^ for anaplerotic pathways [19,20].

Aguilera et al. [18] hypothesized that *ΔCA* is not viable in ambient CO_2_ largely because the cellular HCO_3_^−^ level was insufficient for generating lipids. *S. cerevisiae* uses acetyl-CoA as a building block to synthesize neutral lipids (NL) such as triglycerides (TGs) and sterol-esters (SEs) [21]. The first step in fatty acid biosynthesis is the carboxylation of acetyl-CoA to malonyl-CoA [22]. This reaction uses HCO_3_^−^ generated from CO_2_ by the native CA in the wild-type *S. cerevisiae* cell.

Therefore, *ΔCA* can be used to detect and analyze the activity of normal or modified CAs and unusual CA-like proteins. To test this *ΔCA*-based complementation system, we first used *ΔCA* to investigate the activity of the human CA II (hCA) protein. We then tested the viability of tagged and codon-optimized hCA proteins to see if CA activity is affected by such modifications. We also tested the activity of CAs located in the mitochondria and thylakoids of *C. reinhardtii.* The mitochondrial CAs, CAH4 and CAH5, are β-CAs that are highly expressed in *C. reinhardtii* cells grown in ambient levels of CO_2_ [23,24]. They have been shown to be necessary for optimal photosynthesis in cells grown in limiting-CO_2_ conditions [25]. CAH3 is an α-CA located in the thylakoid lumen of *C. reinhardtii* that generates CO_2_ for fixation by Rubisco inside the pyrenoid [26,27]. We also used an *Arabidopsis thaliana* β-CA called βCA3 to see if the *ΔCA*-based heterologous complementation system works for plant CAs. Aside from viability tests, CA activity was verified using the Wilbur–Anderson assay [28] and isotope exchange membrane-inlet mass spectrometry (MIMS). The results presented in this study suggest that the *ΔCA* strain can be used to determine the activity of CAs from different sources, as well as CAs that have been modified with tags and codon optimization.

## 2. Results

### 2.1. The S. cerevisiae Strain ΔCA Has a High-CO_2_-Dependent Growth Phenotype

**Figure 1:**
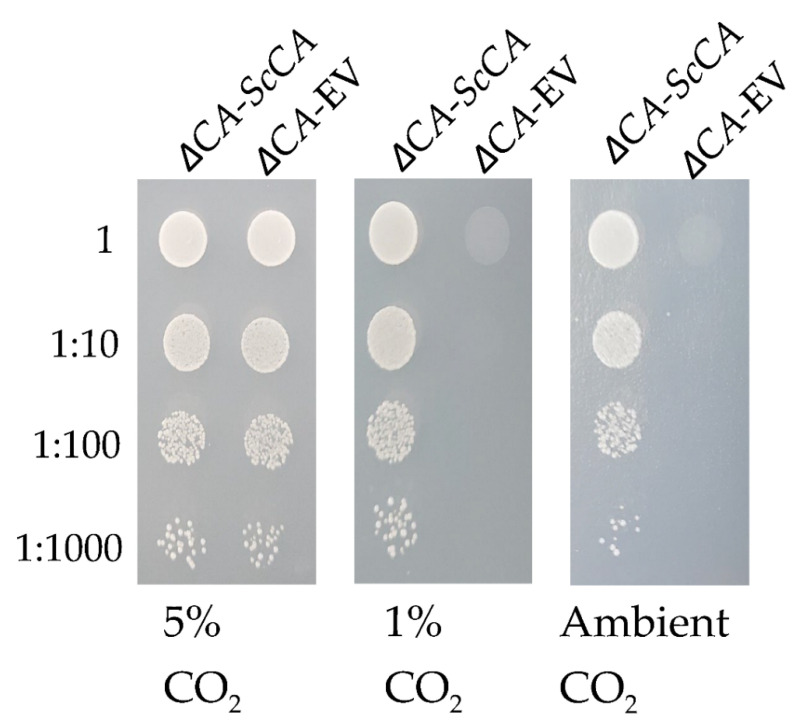
*S. cerevisiae* CA knock-out (*ΔCA*) cannot grow in limiting-CO_2_ conditions. *ΔCA*-EV cells and *S. cerevisiae* CA knock-out out cells complemented with ScCA (*ΔCA*-*ScCA*) were plated in 10 µL spots on YM (-his,-trp) plates and incubated at 30 °C in 5%, 1%, and ambient (0.04%) CO_2_ for 3 days. The cells were standardized to an initial OD_600_ of 0.1 and serially diluted before plating.

**Figure 2:**
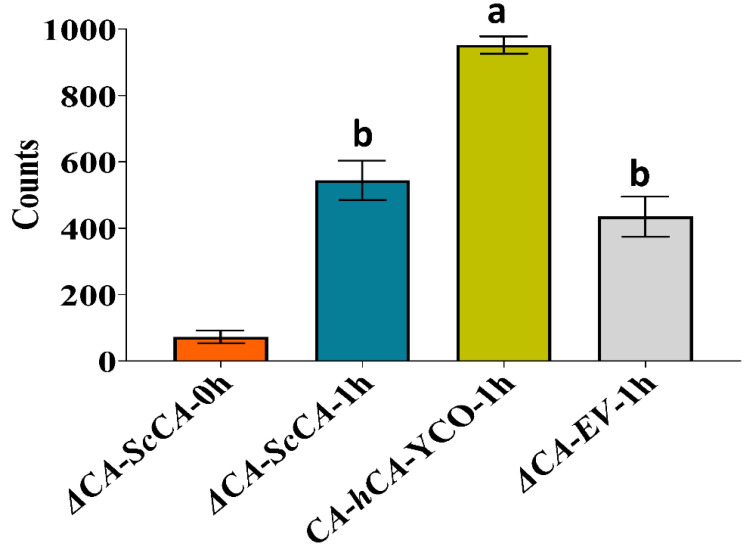
*ΔCA* complemented with yeast codon-optimized (YCO) human CA (*hCA*) incorporates ^14^C-acetic acid into neutral lipids at a faster rate than *ΔCA*-EV and *ΔCA*-*ScCA*. *ΔCA*-*ScCA*, *ΔCA*-*hCA*-YCO, and *ΔCA*-EV cells were grown in liquid YM (-his,-trp) supplemented with ^14^C-acetic acid for one hour in air levels of CO_2_. *ΔCA*-*ScCA* cells supplemented with ^14^C-acetic acid for zero hours is shown as a negative control. ^14^C incorporation was measured using a silicone oil filtering centrifugation assay. Bars represent means, and error bars represent standard errors (*n* = 3). Statistical significance among different groups was computed with ANOVA and Tukey’s post hoc HSD test (*p* < 0.05), and different statistical groups are represented by the letters a and b above bars.

The CO_2_ level requirement of *ΔCA* was characterized by conducting growth assays on solid media supplemented with different levels of CO_2_: 5% (*v*/*v*) CO_2_ in air, 1% (*v*/*v*) CO_2_ in air, and ambient CO_2_ (~0.04% (*v*/*v*) CO_2_ in the air) (Figure 1). For these experiments, *ΔCA*-EV refers to the *ΔCA* strain transformed with an empty vector (EV) containing a selectable gene. For the positive control, *ΔCA* was transformed with a vector containing the *S. cerevisiae NCE103* gene so that it expresses the native CA (*ΔCA*-*ScCA*). Unlike the positive control, *ΔCA* only grows at 5% CO_2_ and dies at 1% and ambient CO_2_. These results agree with an early report by Aguilera et al. [18]. To investigate the biochemical deficiency underlying the high-CO_2_-dependent phenotype of *ΔCA,* we incorporated radiolabeled ^14^C-acetic acid into *ΔCA*-EV, *ΔCA*-*ScCA*, and *ΔCA*-*hCA*-YCO (*ΔCA* complemented with a *S. cerevisiae* codon-optimized (YCO) version of hCA) for one hour in ambient-CO_2_ conditions and assayed ^14^C incorporation into lipids using a silicone oil filtering centrifugation assay. The incorporation of radiolabeled ^14^C in neutral lipids was higher in cells reconstituted with hCA compared to *ΔCA*-EV after one hour. Furthermore, *ΔCA*-*hCA*-YCO had twice the incorporation of ^14^C in neutral lipids compared to *ΔCA* after one hour (Figure 2).

### 2.2. ΔCA Can Be Used as a Heterologous Complementation System to Detect CA Activity of Normal and Modified CAs

**Figure 3:**
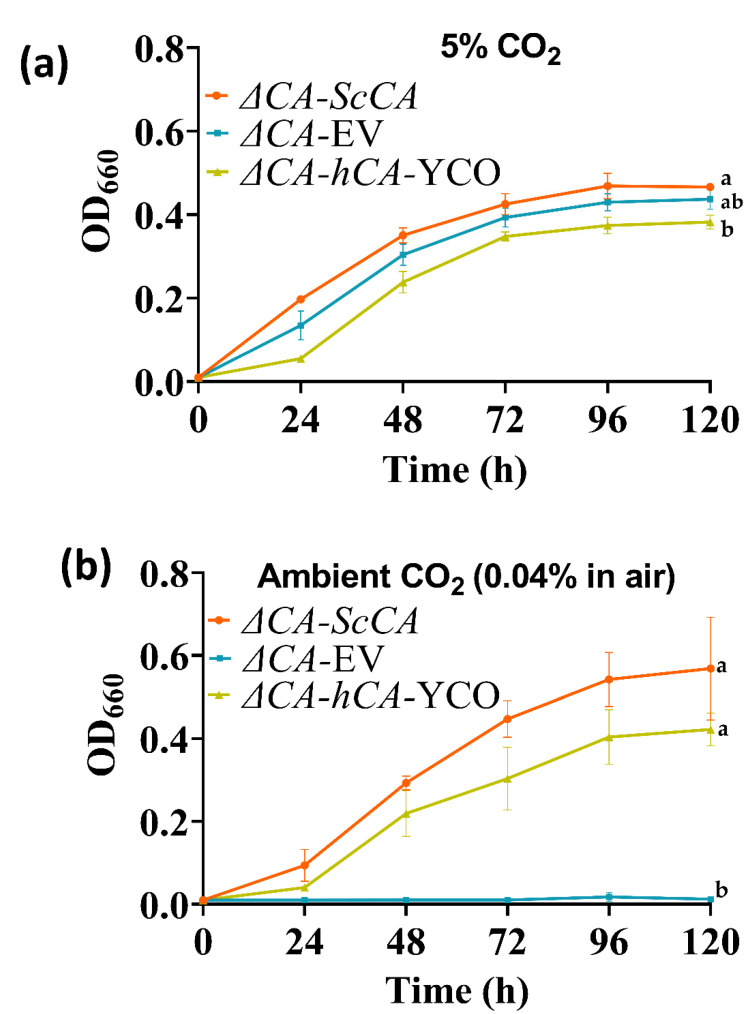
*ΔCA*-*hCA*-YCO can grow in limiting-CO_2_ conditions in liquid media, similar to *ΔCA*-*ScCA*. *ΔCA*-*ScCA*, *ΔCA*-EV, and *ΔCA*-*hCA*-YCO cells were grown in liquid YM (-his,-trp) and incubated at 30 °C in (**a**) 5% and (**b**) ambient CO_2_ for 120 h. The cultures were standardized to an initial OD_600_ of 0.01 in 50 mL. Points in the graph represent means, and error bars represent standard errors (*n* = 3). Statistical significance for the last time point (120 h) was computed with ANOVA and Tukey’s post hoc HSD test (*p* < 0.05), and different statistical groups are represented by letters.

**Figure 4:**
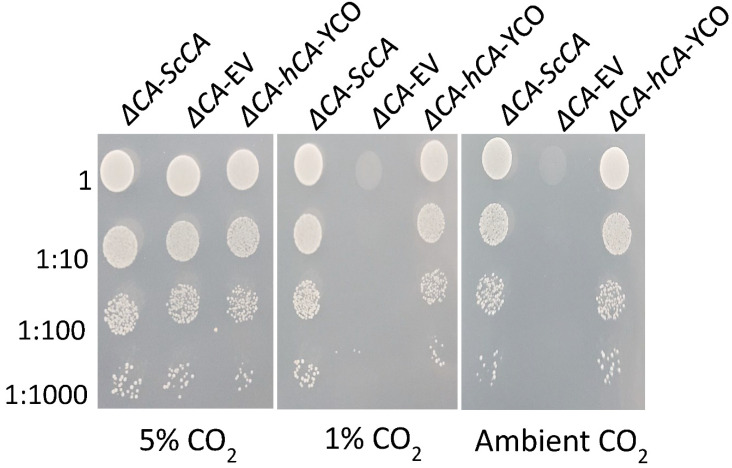
*ΔCA*-*hCA*-YCO can grow in limiting-CO_2_ conditions on solid media, similar to *ΔCA*-ScCA. *ΔCA*-*ScCA*, *ΔCA*-EV, and *ΔCA*-*hCA*-YCO cells were grown to logarithmic phase then plated in 10 µL spots on YM (-his,-trp) plates and incubated at 30 °C in 5%, 1%, and ambient (0.04%) CO_2_ for 3 days. The cells were standardized to an initial OD_600_ of 0.1 and serially diluted before plating.

**Figure 5:**
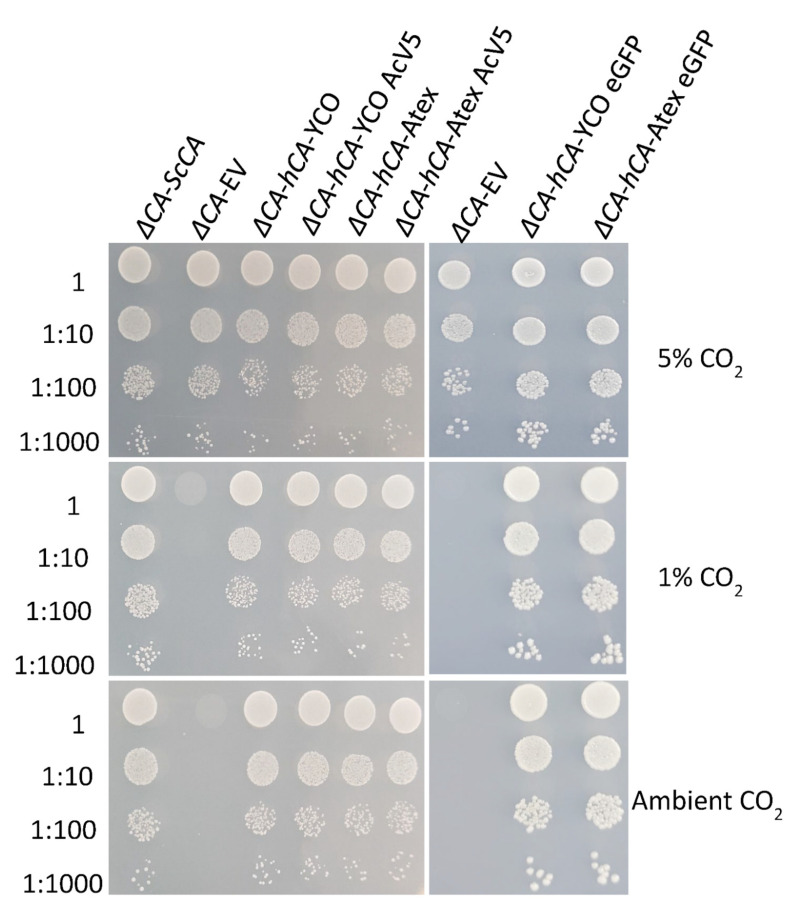
*ΔCA* complemented with modified *hCAs* can grow in limiting-CO_2_ conditions. Modified *ΔCA*-*hCA* constructs still grow in limiting-CO_2_ conditions regardless of codon optimization or added tags. *ΔCA*-*ScCA*, *ΔCA*-EV, *ΔCA*-*hCA*-YCO, *ΔCA*-*hCA*-YCO AcV5, *ΔCA*-*hCA*-YCO eGFP, *ΔCA*-*hCA*-Atex, *ΔCA*-*hCA*-Atex AcV5, and *ΔCA*-*hCA*-Atex eGFP cells were grown to logarithmic phase then plated in 10 µL spots on YM (-his,-trp) plates and incubated at 30 °C in 5%, 1%, and ambient (0.04%) CO_2_ for 3 days. The cells were standardized to an initial OD_600_ of 0.1 and serially diluted before plating.

**Figure 6:**
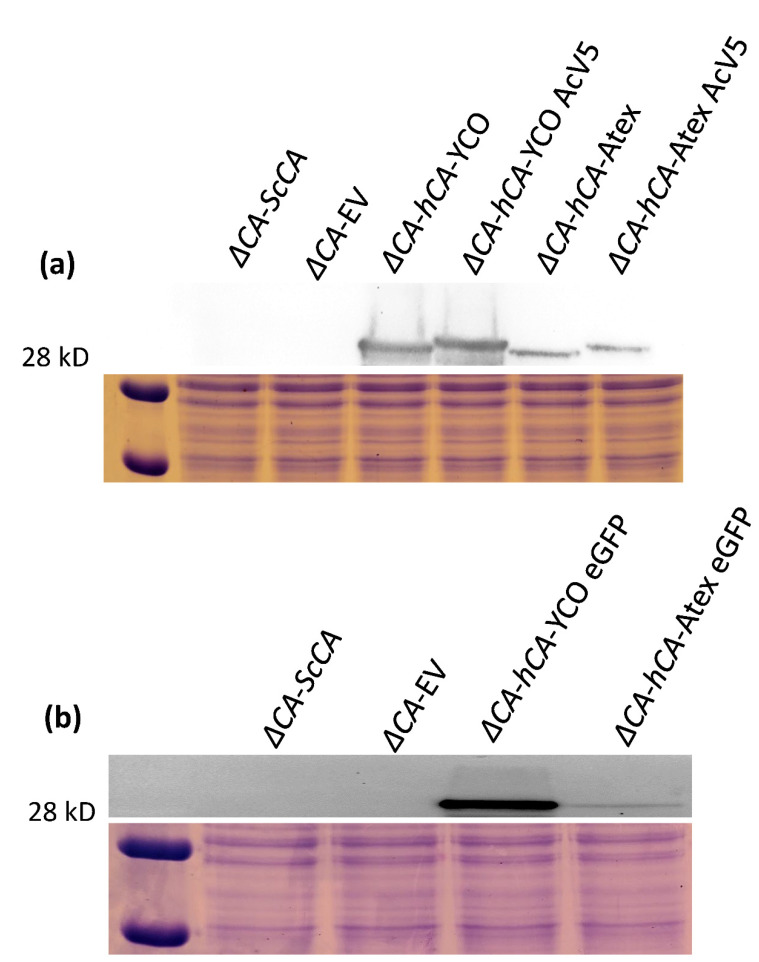
Immunological detection of modified hCAs in *ΔCA*. (**a**) Immunoblot showing hCA expression in *ΔCA*-*ScCA*, *ΔCA*-EV, *ΔCA*-*hCA*-YCO, *ΔCA*-*hCA*-YCO AcV5, *ΔCA*-*hCA*-Atex, and *ΔCA*-*hCA*-Atex AcV5. (**b**) Immunoblot showing hCA expression in *ΔCA*-*ScCA*, *ΔCA*-EV, *ΔCA*-*hCA*-YCO eGFP, and *ΔCA*-*hCA*-Atex eGFP. Cells were grown in liquid YM (-his,-trp) and 5% CO_2_ for 72 h prior to extracting protein. Below the immunoblots are SDS-Page gels loaded with the same protein samples and stained with Coomassie Blue.

The CO_2_ growth requirement of *ΔCA* was used to characterize the CA activity of normal and modified CAs. hCA complemented the *ΔCA* phenotype in ambient CO_2_ and 1% CO_2_ (Figure 3 and Figure 4) [29]. In the liquid growth assay, we observed that *ΔCA*-*hCA*-YCO grew faster than *ΔCA*-EV in ambient CO_2_. However, in both conditions, *ΔCA*-*ScCA* growth was faster compared to *ΔCA*-EV (Figure 3a,b). In the growth assay on solid media, *ΔCA*-*hCA*-YCO grew at a rate similar to *ΔCA*-*ScCA* in ambient CO_2_ and 1% CO_2_ (Figure 4). Next, we transformed *ΔCA* with YCO hCA and Arabidopsis codon-optimized (Atex) hCA and checked their effect on CA activity. Additionally, we added the tags AcV5 and eGFP to *ΔCA*-*hCA*-YCO and *ΔCA-hCA*-Atex to see if they affected the growth of the *S. cerevisiae*. The growth assays on solid media show that the modified hCA variants complemented *ΔCA* in ambient CO_2_ and 1% CO_2_ (Figure 5). We also compared the expression of hCA in the complemented lines by analyzing the protein’s abundance via Western blots. In the strains complemented with the YCO genes (*ΔCA*-*hCA*-YCO, *ΔCA*-*hCA*-YCO AcV5, and *ΔCA*-*hCA*-YCO eGFP), hCA expression was higher compared to strains complemented with Atex genes (*ΔCA*-*hCA*-Atex, *ΔCA*-*hCA*-Atex AcV5, and *ΔCA*-*hCA*-Atex eGFP; Figure 6b). The protein expression of hCA was not affected by the addition of the AcV5 and eGFP tags in strains complemented with the genes optimized for *S. cerevisiae* and Arabidopsis (Figure 6a,b and Figure S2).

### 2.3. Plant and Algal Carbonic Anhydrases Show CA Activity in ΔCA-Based Heterologous Complementation System

**Figure 7:**
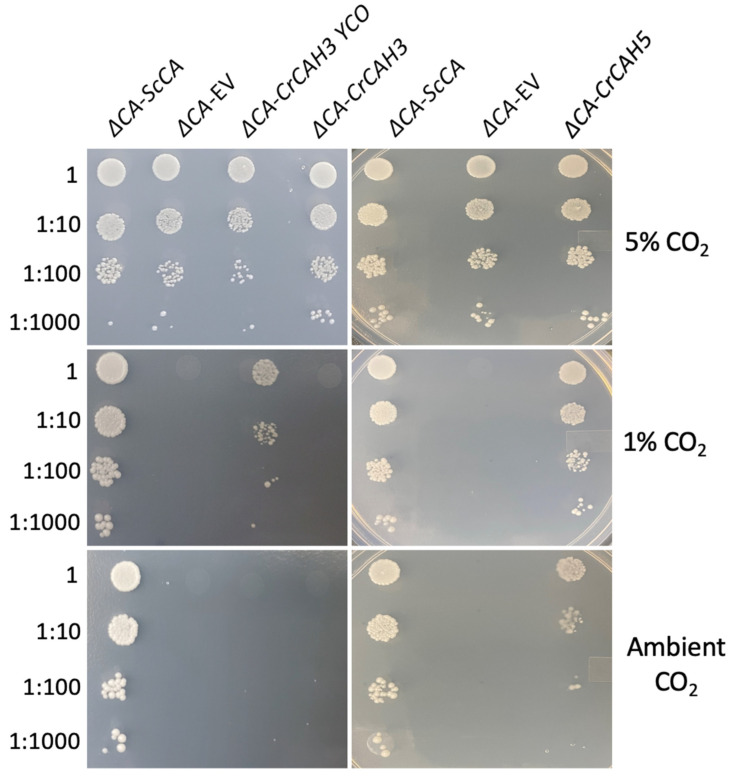
*ΔCA* complemented with *CrCAH3* and *CrCAH5* can grow in limiting-CO_2_ conditions. *ΔCA* complemented with *CrCAH3* (YCO) can grow in 1% CO_2_, while *ΔCA* complemented with *CrCAH5* can grow in 1% CO_2_ and ambient CO_2_. *ΔCA-ScCA*, *ΔCA*-EV, *ΔCA*-*CrCAH3*-YCO, *ΔCA*-*CrCAH3,* and *ΔCA*-*CrCAH5* cells were grown to logarithmic phase then plated in 10 µL spots on YM (-his,-trp) plates and incubated at 30 °C in 5% CO_2_, 1% CO_2_, and ambient CO_2_ (0.04%) for 3 days. The cells were standardized to an initial OD_600_ of 0.1 and serially diluted before plating.

**Figure 8:**
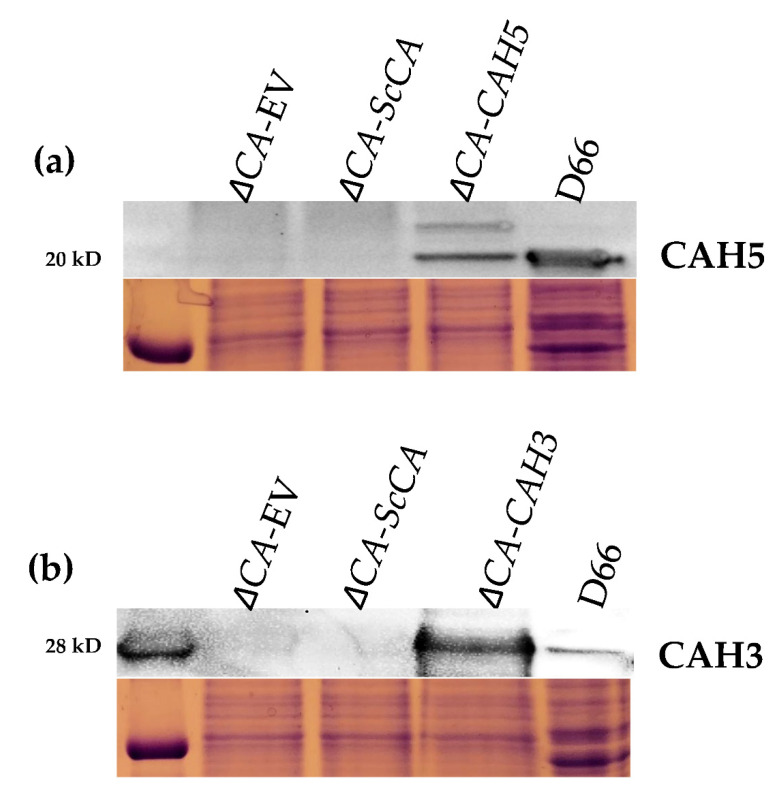
Immunological detection of CrCAH5 and CrCAH3 in *ΔCA*. (**a**) Immunoblot showing CrCAH5 expression in *ΔCA-*EV, *ΔCA*-*ScCA*, *ΔCA*-*CrCAH5,* and wild-type *C. reinhardtii* cells (D66). (**b**) Immunoblot showing CrCAH3 expression in *ΔCA*-EV, *ΔCA*-*ScCA*, *ΔCA*-*CrCAH3*, and wild-type *C. reinhardtii* cells (D66). *S. cerevisiae* cells were grown in liquid YM (-his,-trp) and 5% CO_2_ for 72 h prior to extracting protein. *C. reinhardtii* cells were grown in MIN media and low CO_2_ conditions for 12 h prior to extracting protein. Below the immunoblots are SDS-Page gels loaded with the same protein samples and stained with Coomassie Blue.

To test the hypothesis that the *ΔCA*-based heterologous complementation system can rapidly detect the activity of different algal CA isoforms, we expressed the *C. reinhardtii* β-carbonic anhydrase CAH5 and α-carbonic anhydrase CAH3 in *ΔCA.* Expression of CAH5 restored a normal growth phenotype in the *ΔCA* mutant when cells were grown in ambient CO_2_ and 1% CO_2_ (Figure 7). CAH5 protein expression was detected in *ΔCA*-*CrCAH5* and in the positive control D66 (a wild-type *C. reinhardtii* strain) (Figure 8a). The full-length coding sequence (CDS) was used for the expression of CAH5 in *ΔCA.* The Western blot shows that *S. cerevisiae* was able to process the N-terminal mitochondrial sequence of CAH5 (Figure 8a). We observed a full-length polypeptide of 27.8 kD and a cleaved polypeptide of 20.4 kD, which is similar to the size observed in the positive control. This is the first report showing that CAH5 is an active CA in a heterologous system. The expression of CAH3 in *ΔCA*-*CrCAH3*-YCO restored growth in 1% CO_2_, but no growth was observed in ambient CO_2_ (Figure 7). In the *ΔCA*-*CrCAH3* strain containing the native *C. reinhardtii CAH3* gene, growth was not restored in either limiting-CO_2_ condition. The Western blot shows CAH3 expression in *ΔCA*-*CrCAH3*-YCO and in the positive control (Figure 8b).

**Figure 9:**
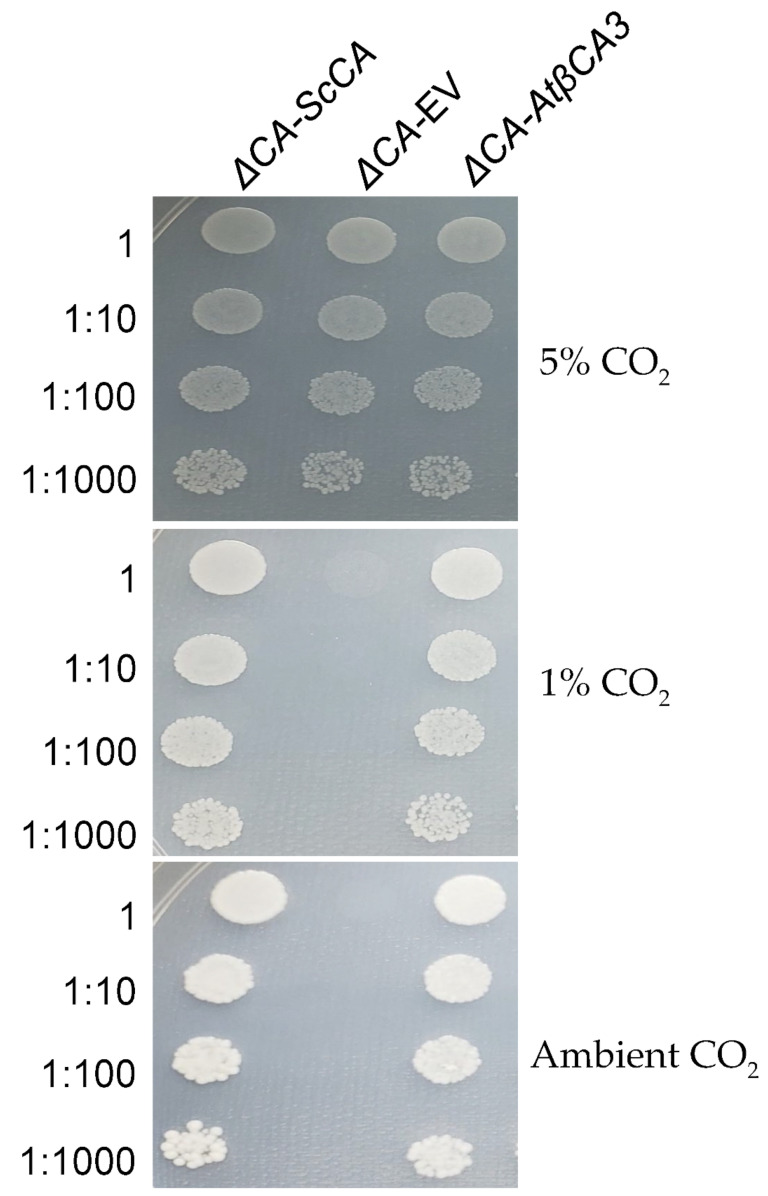
*ΔCA* complemented with *AtβCA3* can grow in limiting-CO_2_ conditions. *ΔCA*-*ScCA*, *ΔCA*-EV, and *ΔCA*-*AtβCA3* cells were grown to logarithmic phase then plated in 10 µL spots on YM (-his,-trp) plates and incubated at 30 °C in 5% CO_2_, 1% CO_2_, and ambient CO_2_ (0.04%) for 3 days. The cells were standardized to an initial OD_600_ of 0.01 and serially diluted before plating.

To test the activity of plant carbonic anhydrases in *ΔCA*, we transformed the *S. cerevisiae* mutant with the cytosolic carbonic anhydrase βCA3 from Arabidopsis. Normal growth was observed in *ΔCA*-*AtβCA3* at all three CO_2_ levels (Figure 9). This result suggests that βCA3 is an active CA.

### 2.4. ΔCA-Based Heterologous Complementation System Can Be Used to Quantify CA Activity Using Wilbur–Anderson Assay and MIMS

**Figure 10:**
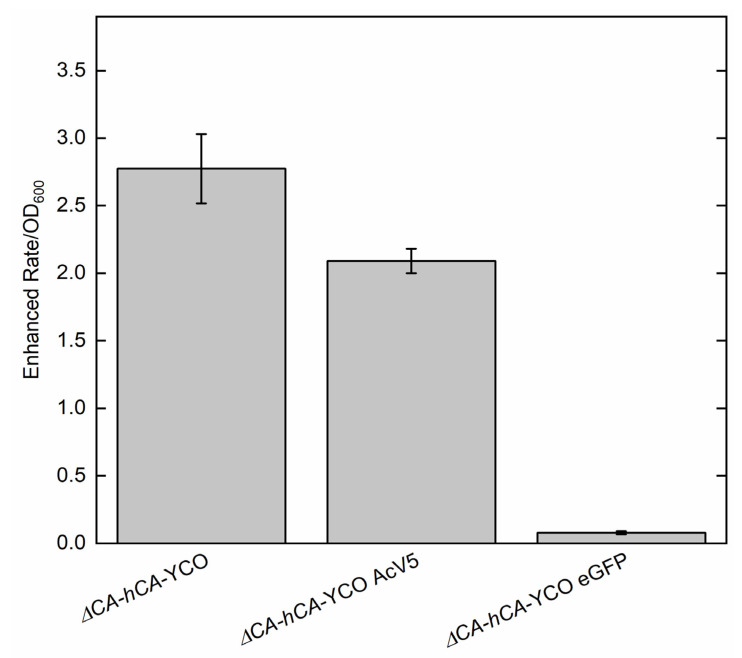
MIMS can be used to measure CA activity in *ΔCA* strains. MIMS assays were performed at 25 °C and pH 7.4 on protein extracts from *ΔCA*-*hCA*-YCO, *ΔCA*-*hCA*-YCO AcV5, and *ΔCA*-*hCA*-YCO eGFP. All strains were grown in ambient CO_2_ for 48 h. The enhanced rate of ^18^O exchange between ^13^C^18^O_2_ and H_2_^16^O was calculated as the ratio between the catalyzed rate of ^18^O loss from ^13^C^18^O_2_ and the uncatalyzed rate in the absence of CA. Columns and error bars represent the mean ± standard deviation of three technical replicates for each *S. cerevisiae* strain.

To rapidly quantify the activity of normal or modified CAs in the *ΔCA* system, we used the Wilbur–Anderson assay and MIMS. The Wilbur–Anderson assay was successful in demonstrating the CA activity of *ΔCA* strains complemented with different variants of hCA (Table 1). CA activity was recorded as 4.7 ± 0.5 WAU mg^−1^ in *ΔCA*-*hCA*-YCO, which was the highest of all tested strains. The addition of AcV5 and eGFP decreased the CA activity to 3.9 ± 0.4 WAU mg^−1^ and 2.5 ± 0.3 WAU mg^−1^, respectively. In the strains using Atex genes, CA activity was further reduced. The strain *ΔCA*-*hCA*-Atex showed CA activity around 1.9 ± 0.2 WAU mg^−1^. Similar to the tagged YCO strains, the addition of AcV5 and eGFP decreased CA activity to 0.9 ± 0.1 WAU mg^−1^ and 1.4 ± 0.2 WAU mg^−1^, respectively. CA activity was also measured in the *ΔCA* strains using MIMS (Figure 10). We found that the cell lysate in *ΔCA*-*hCA*-YCO exhibited maximum CA activity. The AcV5 and eGFP tags reduced the CA activity significantly, consistent with measurements obtained using the Wilbur–Anderson assay.

## 3. Discussion

In this report, an *S. cerevisiae* CA knock-out strain (*ΔNCE103* or *ΔCA*) was utilized as a successful heterologous system for screening active carbonic anhydrases from plants and algae. Additionally, *ΔCA* has previously been reported as a potential tool for accelerating the discovery of non-sulfonamide-based CAIs (carbonic anhydrase inhibitors) for the treatment of CA-related diseases, such as glaucoma [30].

In this study, *ΔCA* was generated and found to have a high-CO_2_-dependent phenotype, meaning it requires high-CO_2_ conditions to survive (Figure 1). This clear high CO_2_ growth requirement indicates that *ΔCA* strains can be used for fast and accurate screening of CA activity or active bicarbonate transport. To confirm this hypothesis, we tested CAs from human red blood cells (hCA), Arabidopsis, and *C. reinhardtii* in the heterologous *S. cerevisiae* system. Along with rapidly screening for CA activity, *ΔCA* can also be used for the estimation of CA enzymatic activity using the Wilbur–Anderson assay and MIMS.

In mammals, CAs are expressed in almost all tissues and are involved in oxygen transport between lungs, red blood cells and tissues; pH regulation; ion exchange in the kidney; and electrical activity in the retina and nervous system [31,32,33]. Autotrophic organisms use CAs in CCMs, where CAs are involved in increasing inorganic carbon for carbon fixation [3]. In contrast, very little is known about the physiological role of CAs in heterotrophic microbes. According to Aguilera et al. [18], the CA-deficient *S. cerevisiae* mutant’s need for elevated CO_2_ concentrations originates from three bicarbonate-dependent carboxylation reactions catalyzed by pyruvate decarboxylase, acetyl-CoA carboxylase, and carbamoyl phosphate synthetase. These enzymes are involved in the synthesis of C_4_ intermediates, fatty acids, arginine, and uracil, respectively [34]. These observations demonstrate that the *S. cerevisiae* CA is a key biosynthetic enzyme responsible for the viability of *S. cerevisiae* under aerobic conditions. Since *ΔCA* was complemented at air levels of CO_2_ by the addition of hCA (Figure 3 and Figure 4), we investigated whether fatty acid biosynthesis is bicarbonate-dependent at air levels of CO_2_ (Figure 2). *S. cerevisiae* uses acetyl-CoA as a building block to synthesize neutral lipids (NL) such as triglycerides (TGs) and sterol-esters (SEs). Acetyl-CoA is first converted into malonyl-CoA by acetyl-CoA carboxylase, using HCO_3_^−^ as a substrate. *S. cerevisiae* cells deficient in acetyl-CoA carboxylase are not able to make long chain saturated fatty acids for de novo growth [35]. To test our hypothesis, we introduced ^14^C-acetic acid to *ΔCA*-*ScCA*, *ΔCA*-EV, and *ΔCA*-*hCA* grown on air levels of CO_2_ for one hour. Acetic acid is rapidly converted into acetyl-CoA by acetyl-CoA synthetase (ACS2), which makes ^14^C-acetic acid, a suitable radiolabeling substrate in *S. cerevisiae*. We observed that radiolabeled ^14^C is incorporated into the chloroform–methanol fraction containing NLs. The incorporation of radiolabeled ^14^C into NLs occurs at a higher count in *ΔCA*-*hCA* than in *ΔCA*-*ScCA* and *ΔCA-*EV (Figure 2). This result confirmed that CAs produce HCO_3_^−^ for the NL biosynthesis pathway. However, there are other bicarbonate-requiring pathways that are also involved in limiting the growth of *ΔCA* in air. Hence, if we introduce CAs or HCO_3_^−^ transporters from plants and algae, they can help to increase the HCO_3_^−^ pool required for different biological processes in the cell. This makes the *ΔCA* heterologous complementation system useful for identifying new bicarbonate transporters or CAs as suitable candidates to improve photosynthetic efficiency in C_3_ crop plants.

This report shows that hCA displayed sufficient CA activity to rescue *ΔCA* grown on air levels of CO_2_ (Figure 3 and Figure 4). The results extend the work of Sangkaew et al. [30] who used this *ΔCA* system to screen CA inhibitors. To test modified CAs, *S. cerevisiae* codon-optimized (YCO) and Arabidopsis codon-optimized (Atex) hCA genes with added eGFP and AcV5 tags were used to complement *ΔCA* (Figure 5. The hCA protein was detected in all the hCA variants, but the amount of protein was highest in *S. cerevisiae* codon-optimized hCA (Figure 6a,b). The different variants of hCA complement *ΔCA* even though the protein content differs in the strains. This relates to the concept that only a low amount of CA activity is needed to maintain vital biological functions in *S. cerevisiae* since CA is such a fast enzyme. This concept is also supported by plant studies showing that the majority of CA activity within the plant needs to be removed in order to observe a growth phenotype [10]. Although all the hCA variants rescued *ΔCA*, CA activity was highest in the strain using *S. cerevisiae* codon-optimized hCA (Table 1 and Figure 10). The low CA activity in Atex strains might also be because of low protein expression (Figure 6a,b). In terms of the effect of added tags, the MIMS and Wilbur–Anderson assay data showed that the addition of longer tags resulted in a larger reduction in CA activity. The hCA tagged with eGFP has lower CA activity compared to the hCA tagged with AcV5 (Table 1 and Figure 10). These results suggest that the *ΔCA* system can be used to rapidly test the suitability of carbonic anhydrases before introducing them into C_3_ plants_._ Tags such as eGFP and AcV5 are widely used to determine the subcellular location of CAs, but there is a possibility that these tags can affect the functionality of the proteins. Thus, the *ΔCA*-based heterologous complementation system provides a rapid pipeline for the systematic assessment of normal and modified CAs before introducing them into C_3_ plants. Mathematical models predict that installing a CCM into C_3_ plants could improve leaf CO_2_ uptake by up to 60% [36,37]. Hence, using *ΔCA* in conjugation with the Wilbur–Anderson assay and MIMS can shorten the process of selecting suitable CCM components from cyanobacteria and algae to transform into terrestrial C_3_ plants.

To check the CA activity from an algal system, we expressed the β-carbonic anhydrase, CAH5, and the α-carbonic anhydrase, CAH3, from *C. reinhardtii* in *ΔCA.* CAH5 is present in the mitochondrial matrix and is required to maintain optimal rates of photoautotrophic growth on ambient levels of CO_2_ [25]. CAH5 restores the growth of *ΔCA* at air levels of CO_2_ and 1% CO_2_, but the rescued phenotype is weak compared to *ΔCA*-*ScCA* (Figure 7). This might indicate that CAH5 is a low-activity CA. Mitochondrial CA in *C. reinhardtii* is encoded by two genes (*CAH4* and *CAH5*). This genetic redundancy could help the cell increase the amount of CA in the mitochondria since the enzyme has low activity, but it seems wasteful to produce large amounts of a protein with low activity rather than making a protein with high activity. The α carbonic anhydrase CAH3 was also tested in *ΔCA.* It is located in the thylakoid lumen in *C. reinhardtii* and has also been identified as an important component to maintain the CCM at low CO_2_ levels [38,39]. It was previously reported that CrCAH3 is different from other α carbonic anhydrases given that it has an optimum CA activity at lower pH values than CAs of the same type, which normally operate at pH 7.0 and higher [6,40]. *S. cerevisiae* codon-optimized CAH3 restored the normal growth phenotype in *ΔCA* at 1% CO_2_ but not at air levels, indicating that CAH3 activity might have been reduced because the *S. cerevisiae* cytoplasmic pH is around 7 (Figure 7). The Western blot clearly shows that CrCAH3 is produced in *ΔCA* (Figure 8b). The chloroplast transit peptide was removed from CrCAH3. The low activity could also be attributed to our use of a truncated version of CAH3 in *ΔCA*, although the protein length we used was reported to be the mature protein size [6,26]. Another possibility is that CAH3 requires post-translational modifications. Blanco-Rivero, et al. [41] reported that kinase activity is needed to activate CAH3 inside the lumen. The complementation of CrCAH5 and CrCAH3 (both YCO) in *ΔCA* suggests that the *ΔCA*-based heterologous complementation system can detect active CA enzymes from algal systems.

In general, α-carbonic anhydrases are structurally simpler than β-carbonic anhydrases and often have high specific activity. Humans only have α-carbonic anhydrases, while plants and algae have a wide variety of carbonic anhydrase families from α, β, γ, and θ classes. These CAs are localized to different intercellular and intracellular locations. Hence, the *ΔCA* heterologous complementation system could also be used as a tool to differentiate activity between CA families, which could help researchers select better CA candidates to integrate into C_3_ crop plants.

To check if *ΔCA* is rescued by a plant’s CA, we used β carbonic anhydrase βCA3, which localizes in the cytosol of Arabidopsis. βCA3 rescued *ΔCA* on 1% CO_2_ and air levels of CO_2_, showing that it is an active CA enzyme (Figure 9).

**Figure 11:**
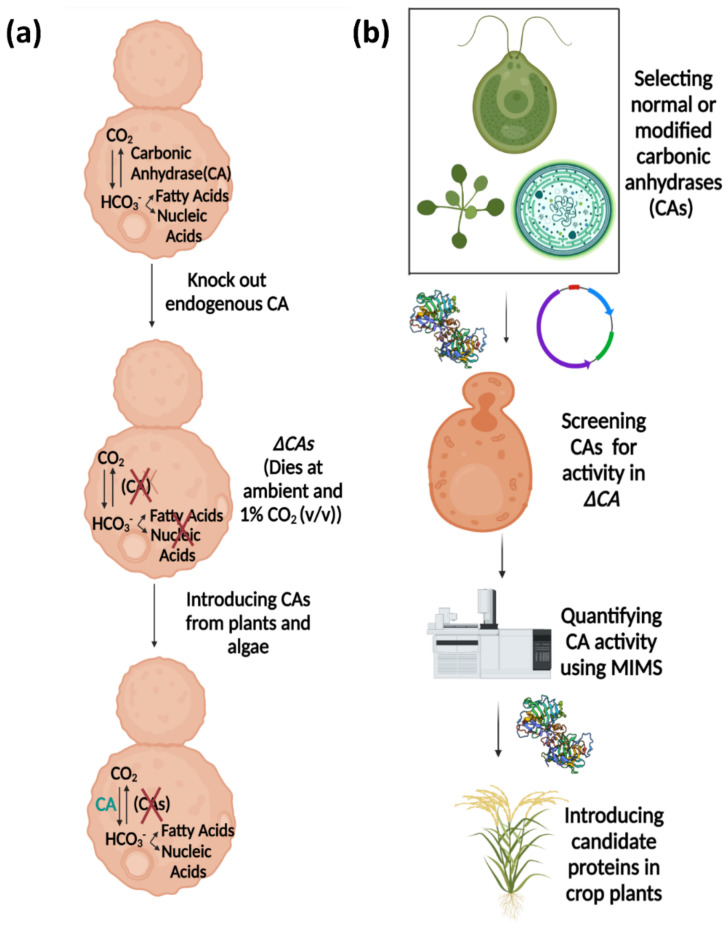
Model proposing the use of *ΔCA-*based heterologous complementation systems to detect CA activity. In (**a**) the importance of carbonic anhydrase (CA) for the growth of *S. cerevisiae* at ambient CO_2_ is shown and (**b**) discusses the pipeline to use *ΔCA* as a useful tool for rapidly detecting CA activity before introducing normal or modified CAs in crop plants.

After transforming crop plants with a protein, the main challenge occurs upon trying to determine if the protein is functional once it is correctly localized. The addition of a fluorescent tag reveals if the protein is in the correct location, but it cannot report if the protein is in the correct orientation in the membrane or if it is functional. In addition, when GFP is added to a protein, the protein is modified because an entire second protein has been attached. This modification could potentially alter the CAs’ activity. The heterologous *ΔCA*-based system is an important tool for rapidly checking the activity of normal or modified CAs before integrating them into C_3_ crop plants to improve photosynthetic efficiency (Figure 11a,b). The *ΔCA* system can also check the activity of proteins retargeted to different compartments in plants.

## 4. Materials and Methods

### 4.1. S. cerevisiae Strain and Growth Conditions

*Saccharomyces cerevisiae* strain DDY2 (*S. cerevisiae* W303-1a diploid variant) was used as the starting stock for the generation of a CA knock-out (*ΔNCE103*). Yeast minimal media (YM) supplemented with 6.7 g/L yeast nitrogen base, 20% (*w*/*v*) dextrose, and an amino acid mix was used to grow liquid cultures of the *S. cerevisiae* cells. YM plates were made by adding 1.5% (*w*/*v*) agar to liquid YM. The *ΔCA* generated in this study was grown in 5% (*v*/*v*) CO_2_ in air at 30 °C, unless otherwise stated. Liquid cultures were grown on a rotary shaker at 30 °C in 5% (*v*/*v*) CO_2_ in air and ambient CO_2_ (0.04% (*v*/*v*) CO_2_ in air). *S. cerevisiae* cells were grown on YM plates in three different CO_2_ conditions: 5% (*v*/*v*) CO_2_ in the air, 1% (*v*/*v*) CO_2_ in the air, and ambient CO_2_ (0.04% (*v*/*v*) CO_2_ in the air) at 30 °C. Where applicable, amino acid mixes were added in the following order for the strains generated in this report: amino acid mix made without tryptophan (*ΔCA*), amino acid mix made without histidine and tryptophan (*ΔCA-*EV, *ΔCA*-*ScCA*, *ΔCA*-*hCA*-YCO, *ΔCA*-*hCA*-YCO AcV5, *ΔCA*-*hCA*-Atex, *ΔCA*-*hCA*-Atex AcV5, *ΔCA*-*CrCAH5*, *ΔCA*-*CrCAH3-*YCO, *ΔCA*-*CrCAH3*, and *ΔCA*-*AtβCA3*) and amino acid mix made without uracil and tryptophan (*ΔCA*-*hCA*-YCO eGFP, *ΔCA*-*hCA*-Atex eGFP).

### 4.2. Generation of S. cerevisiae Carbonic Anhydrase Knock-Out

The construction of the *NCE103* deletion in the diploid strain DDY2 was carried out by PCR-targeting with a *TRP1 disruption cassette* flanked by short homology regions of the *NCE103* gene [42]. The disruption cassette was obtained by amplifying the TRP1 cassette from the plasmid pRS304 ([43] and Table S1). pRS304 was used as a template in a PCR reaction to amplify TRP1 with *NCE103* flanking sequences using oligonucleotides DDO-1976 and -1977. This DNA was concentrated by ethanol precipitation and transformed into *S. cerevisiae* strain DDY2. The transformation mix was plated onto minimal media lacking tryptophan to screen for *ΔNCE103* mutants. The successful haploid knock-outs were confirmed by PCR using primers described in Table S1. Correctly targeted strains were sporulated to haploid, and Trp+ isolates were re-confirmed by PCR as *ΔNCE103* mutants.

### 4.3. Genetic Constructs and Vectors

To express the mature peptide versions of human CAII (HCAII; Genbank ID AK312978) and *CrCAH3*-YCO (referred to as yeast codon-optimized (YCO)) in *ΔNCE103*, the *hCA* and *CrCAH3* gene was synthesized by GenScript in pENTR and cloned into destination vectors MGO515 (-HIS) and MGO528 (-URA) using Gateway cloning. The *hCA* gene was codon optimized for *S. cerevisiae* (referred to as yeast codon-optimized (YCO)) and Arabidopsis (referred to as Arabidopsis codon-optimized (Atex)). A C-terminal AcV5 tag and eGFP tag were added to the *hCA* gene (Figure S1). *hCA* (with or without *Acv5* tag) coding sequences were commercially synthesized (Genscript) as gateway-enabled entry vectors (i.e., included flanking attL sites). A second set of *hCAII* genes without the stop codon were also made, for use in GFP C-terminal fusion constructs. The *hCAII* coding sequences (CDS) were cloned into plasmids from the Advanced Gateway^®^ adapted pRS series of yeast expression plasmids [44] using Gateway *LR Clonase II* enzyme mix (Invitrogen™)—essentially swapping out the ccdB bacterial lethality cassette for the given *hCAII* CDS between the attR1/R2 sites (Figure S1). The yeast expression construct library was obtained through Addgene (https://www.addgene.org/) (kit #1000000011). pAG423GPD-ccdB (internally designated MG0515; HIS) was used for full-length CDS clones. pAG426GPD-ccdB-eGFP (internally designated MG0528; URA) was used for GFP fusions (i.e., *hCAII* CDS without stop codon). All *E. coli* cloning steps used One Shot™ OmniMAX™ (Peachtree Corners, GA, USA) 2 T1R Chemically Competent E. coli cells (Invitrogen). All final plasmids were sanger sequenced to confirm accuracy of the clones using Wizard^®^ Plus SV Minipreps DNA Purification Systems (Promega, Madison, WI, USA), BigDye^®^ sequencing chemistry (Thermofisher Scientific, Waltham, MA, USA), and ZR DNA Sequencing Clean-Up Kit (Zymo Research, Irvine, CA, USA). Internal catalogue designations for final yeast *hCAII* expression constructs were: MG0515.54: GPD-hCAII (YCO)-stop; MG0515.55: GPD-hCAII (YCO)-AcV5-stop; MG0515.56: GPD-hCAII (Atex)-stop; MG0515.57: GPD-hCAII (Atex)-AcV5-stop; MG0528.20: GPD-hCAII (YCO)-nostop-eGFP; MG0528.21: GPD-hCAII (Atex)-nostop-eGFP.

The genes *CrCAH5*, *CrCAH3*, *ScCA*, and *AtβCA3* were amplified by PCR from *C. reinhardtii* (D66) and *A. thaliana* (Col-0). The genes were cloned into the expression vector pDD506 using ClaI/XhoI sites for constitutive expression under control of the *ADH1* promoter (Figure S1). All DNA constructs were verified by DNA sequencing.

### 4.4. S. cerevisiae CA Knock-Out Transformation

The plasmid MG0515 containing *hCA*-YCO, *hCA*-YCO AcV5, *hCA*-Atex, *hCA*-Atex AcV5, and *CrCAH3*-YCO, the plasmid MGO528 containing hCA-YCO eGFP, and *hCA*-Atex eGFP, and the plasmid pDD506 containing *CrCAH5*, *CrCAH3*, and *AtβCA3* were transformed in *E. coli* TOP10 cells (One Shot™ TOP10 Chemically Competent *E. coli*). The plasmids were extracted from the transformed *E. coli* cultures using a GeneJET Plasmid Miniprep Kit (Thermo Scientific™) according to manufacturer’s instructions. The plasmids were transformed in the *ΔNCE103* mutant using a *S. cerevisiae* transformation protocol as described by Gietz and Schiestl [45]. The positive colonies were screened by colony PCR using primers complementary to the genes (Table S1).

### 4.5. High-CO_2_-Dependence Growth Assay

*S. cerevisiae* cell cultures were initiated from −80 °C glycerol stocks. The liquid cultures were grown to log phase in liquid YM in 5% CO_2_ at 30 °C. The cultures were reinoculated for the growth assay and grown in 5% CO_2_ and ambient CO_2_. The optical density at 600 nm (OD_600_) of the cultures was adjusted to an initial OD_600_ of 0.01. Relative growth rates were measured in liquid YM by monitoring the cultures’ OD_600_ using a spectrometer (Thermo Fisher Scientific, Waltham, MA, USA). For the measurement of relative growth rates on solidified YM, the OD_600_ of the cultures was standardized to 0.01, and 10 µL of serial dilutions were spotted onto YM plates. The plates were incubated at 30 °C in 5%, 1%, and ambient CO_2_ for 72 h. The plates were photographed after 72 h.

### 4.6. Immunological Detection of Expressed Proteins in S. cerevisiae

Membrane-enriched protein fractions of *S. cerevisiae* were prepared according to Zhang et al. [46]. Proteins from the *S. cerevisiae* *cell* lysate were mixed with 2× Laemmli sample buffer and β-mercaptoethanol and heated to 95 °C for ~7 min prior to resolution by SDS-PAGE on 12% (*v*/*v*) polyacrylamide gels (Mini-PROTEAN TGX, Bio-Rad Laboratories, Hercules, CA, USA). The expression of tagged proteins was detected immuno-chemically after transferring the proteins to a PVDF membrane. The membrane was blocked in TTBS (TBS with 0.1% (*v*/*v*) Tween) with 1% (*w*/*v*) bovine serum albumin at 4 °C overnight. The membrane was treated with monoclonal antibodies for 1 h at room temperature with the following dilutions: 1:1000 for hCA (Sigma-Aldrich, Darmstadt, Germany), 1:20,000 for CrCAH5 (Agrisera, Vännäs, Sweden) and 1:10,000 for CrCAH3 (Agrisera). The anti-CrCAH3 antibody is commercially available at Agrisera and the anti-CrCAH5 antibody was custom made by Agrisera. The membrane was treated with secondary anti-rabbit antibody (HRP conjugated from Bio-Rad) for 1 h at room temperature with the following dilutions: 1:500 for hCA (Sigma-Aldrich, Darmstadt, Germany) and 1:4000 for CrCAH5 and CrCAH3. Antibody binding was visualized by fluorescence detection with Thermo Scientific™ Pierce™ ECL Western Blotting Substrate on a Chemi-Doc XRS (Bio-Rad, Hercules, CA, USA).

### 4.7. Analysis of Neutral Lipid Synthesis Using Radiolabel ^14^C-Acetic Acid

^14^C-acetic acid was incorporated in *S. cerevisiae* using the protocol described by Rogers and Henne [22], except that 50 μL of radiolabeling media was added to 1 mL of cell suspension (final ^14^C-acetic acid concentration = 1.25 μCi/mL). Here, a 1 h radiolabeling pulse incubation was used to label neutral lipid species in *ΔCA*, *ΔCA*-*ScCA* and *ΔCA*-*hCA*-YCO.

### 4.8. Carbonic Anhydrase Activity Assay

CA activity was measured by the Wilbur–Anderson assay according to Mitra et al. [47]. The isotope exchange membrane-inlet mass spectrometry (MIMS) technique was used to measure CA activity as described by Price and Badger [48]. Briefly, 20 μL of *S. cerevisiae* cell lysate was added to a temperature controlled, 2 mL reaction cuvette connected to the inlet of a Finnegan DELTA-V Isotope Ratio Mass Spectrometer (Thermo Fisher Scientific). The 2 mL CA assay consisted of 100 mM HEPES-KOH (pH 7.4), 5 mM dithiothreitol, and ^13^C^18^O_2_. The enhanced rate of ^18^O exchange between ^13^C^18^O_2_ and H_2_^16^O was calculated as a ratio between the increase in ^18^O loss from ^13^C^18^O_2_ in the presence of CA compared to the uncatalyzed rate. Three technical replicates were run at 25 °C for each CA enzyme tested [10].

## Figures and Tables

**Table 1:** The Wilbur–Anderson assay can be used to measure CA activity in the *ΔCA* mutant. The Wilbur–Anderson CA assay was performed on protein extracts from *ΔCA* strains grown in 5% CO_2_ for 72 h. One WAU = (t_0_ − t)/t where t_0_ is the time for the uncatalyzed reaction and t is the time for the enzyme-catalyzed reaction. The negative control (*ΔCA-*EV) has an activity of 0.9 ± 1 WAU mg^−1^.

Biochemical Trait	*ΔCA*-*hCA*-YCO	*ΔCA*-*hCA*-YCO AcV5	*ΔCA*-*hCA*-Atex	*ΔCA*-*hCA*-Atex AcV5	*ΔCA*-*hCA*-YCO eGFP	*ΔCA*-*hCA*-Atex eGFP	*ΔCA*-EV	*ΔCA*-ScCA
**Specific activity**	4.7 ± 0.5 WAU mg^−1^	3.9 ± 0.4 WAU mg^−1^	1.9 ± 0.2 WAU mg^−1^	0.9 ± 0.1 WAU mg^−1^	2.5 ± 0.3 WAU mg^−1^	1.4 ± 0.2 WAU mg^−1^	0.9 ± 0.1 WAU mg^−1^	1.2 ± 0.1 WAU mg^−1^

## Data Availability

All of the new data is contained within this article or the supplementary material.

## Supplementary Materials

The following supporting information can be downloaded at: https://www.mdpi.com/article/10.3390/plants11141882/s1; Figure S1: Plasmids pDD506(HIS3), MGO515(HIS3), and MGO528(URA3) were used in this study for the overexpression of proteins in *S. cerevisiae*, Figure S2: Relative intensity of the modified hCA bands for the immunoblot experiment shown in Figure 6a,b; Table S1: Primers used in this study for cloning.
