# Supplementary material for: A Rapid Method for Detecting Normal or Modified Plant and Algal Carbonic Anhydrase Activity Using Saccharomyces cerevisiae
Source: Plants (Basel). 2022 Jul 20;11(14):1882. doi: 10.3390/plants11141882 (PMC9320139; doi:10.3390/plants11141882)
Supplement: Supplementary file 1 [file plants-11-01882-s001.zip › plants-1796739-supplementary.pdf]

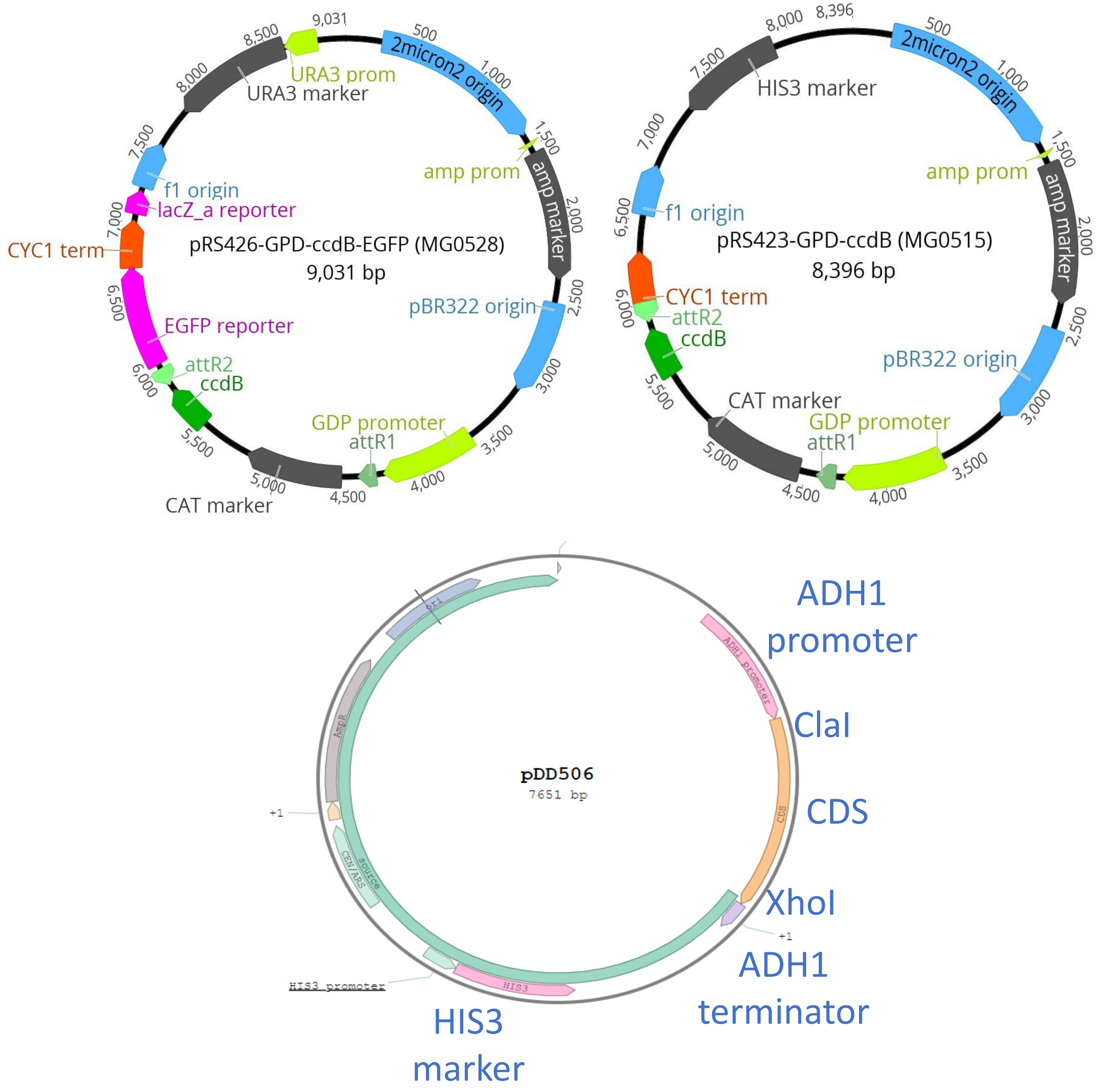

Figure S1. Plasmids MGO515 (HIS3), MGO528 (URA3) and pDD506 (HIS3) were used in this study for the overexpression of proteins in *S. cerevisiae*

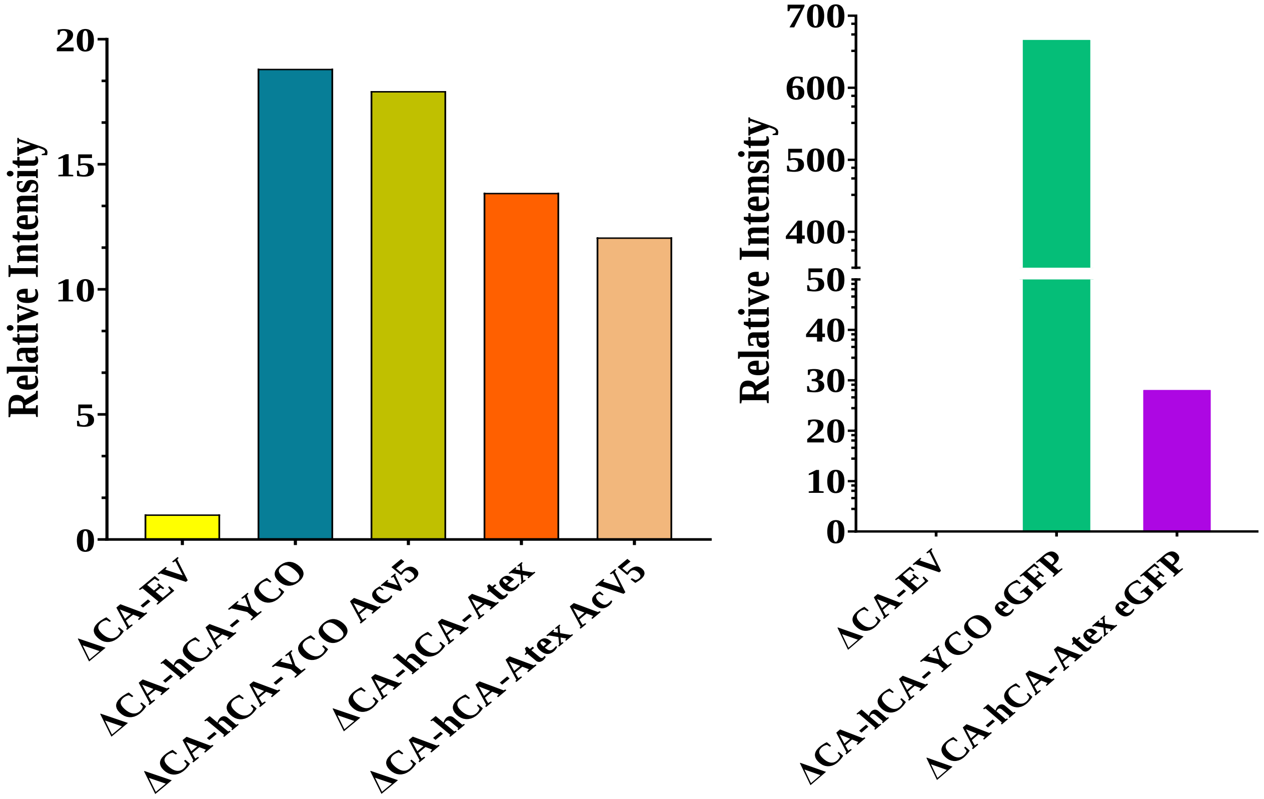

Figure S2: Relative intensity of the modified hCA bands for the immunoblot experiment shown in Figures 6a and 6b

Table S1 . Primers used in this study for cloning.

A. Primers used in the PCR amplified disruption cassettes for gene disruption

Primer Name	Sequence
DD0-1976	TCTTCTGAAAACCCCAACCACATCAACTACAGCTAAGACTACAAATTTCAATT ATTACACATCAGAGCAGATTGTACTGAGAGTGC
DD0-1976	CCGTCTACTTTGTAAATGTCTTTCTATTTCAATGAATATTATATAAGTATATCGGT GAGGCTAAAACTCCTTACGCATCTGTGCGG

B. Primers used for the confirmation of successful gene disruption

Primer Name	Sequence
NCE103 delete check Fw	GAATTAATTCGCATTGTCACCATG
NCE103 delete check Rv	CATCATTCCTATTCAAAAGGTAAG
pRS universal Fw	GCACTCTCAGTACAATCTGC
pRS universal Rv	CCGCACAGATGCGTAAGGAG

C. Primers used for the cloning

Primer Name	Sequence
CAH5 Fw	CTATATCGATATGTCGTCGCGGAATGTCGCT
CAH5 Rv	CTATCTCGAGGCTTAGGCAATCTCGGTCACCTTGC
CAH3 Fw	CTATATCGATATGGCAGCTTGGAACTATGGCG
CAH3 Rv	CTATCTCGAGGCTCGTATTCGACCAGGCGG
AtβCA3 Fw	CTATATCGATATGTCGACAGAGTCGTACG
AtβCA3 Rv	CTATCTCGAGTTAAGACAAGGCAAAGGCA
